# Electrical stimulation as a novel tool for regulating cell behavior in tissue engineering

**DOI:** 10.1186/s40824-019-0176-8

**Published:** 2019-12-05

**Authors:** Cen Chen, Xue Bai, Yahui Ding, In-Seop Lee

**Affiliations:** 1 College of Life Sciences and Medicine, Zhejiang Sci-Tech University, Hangzhou, 310018 People’s Republic of China; 2 Zhejiang Provincial Key Laboratory of Silkworm Bioreactor and Biomedicine, Hangzhou, 310018 People’s Republic of China; 3 Department of Cardiology, Zhejiang Provincial People’s Hospital, Hangzhou, 310014 People’s Republic of China; 4 People’s Hospital of Hangzhou Medical College, Hangzhou, 310014 People’s Republic of China; 5 Institute of Natural Sciences, Yonsei University, 134 Shinchon-dong, Seodaemoon-gu, Seoul, 03722 Republic of Korea

**Keywords:** Electrical stimulation, Tissue engineered materials, Regenerative medicine

## Abstract

Recently, electrical stimulation as a physical stimulus draws lots of attention. It shows great potential in disease treatment, wound healing, and mechanism study because of significant experimental performance. Electrical stimulation can activate many intracellular signaling pathways, and influence intracellular microenvironment, as a result, affect cell migration, cell proliferation, and cell differentiation. Electrical stimulation is using in tissue engineering as a novel type of tool in regeneration medicine. Besides, with the advantages of biocompatible conductive materials coming into view, the combination of electrical stimulation with suitable tissue engineered scaffolds can well combine the benefits of both and is ideal for the field of regenerative medicine. In this review, we summarize the various materials and latest technologies to deliver electrical stimulation. The influences of electrical stimulation on cell alignment, migration and its underlying mechanisms are discussed. Then the effect of electrical stimulation on cell proliferation and differentiation are also discussed.

## Background

Regenerative medicine and tissue engineering are new scientific and technological undertakings of biomedicine that combine aspects of medicine, cell, and molecular biology, materials science and bioengineering, to regenerate, repair or replace tissues or organs [1, 2]. The three key factors of regenerative medicine and tissue engineering include seed cell, scaffold, and stimulating factor [3]. Tissue engineering scaffold could deliver specific cells to the damaged site and as a medium to provide stimuli, with the help of an appropriate structure, similar composition to natural tissue. Scaffold mimic nature tissue’s mechanical properties and desired biological properties, to ensure in vivo support, optimum diffusion of nutrients, and encourage cellular communication [4, 5], which is an indispensable part of tissue engineering. Seed cell include non-stem cells and stem cells. The non-stem cells includes: Schwann cells (SCs), osteoblasts, fibroblast, and endothelial cells (ECs); the stem cells can be divided into two types: (1) adult stem cell: Adipose derived stem cells and muscle derived stem cells; and (2) non-adult stem cell: Embryonic stem cells (ESCs), neural stem cells (NSCs) and bone mesenchymal stem cells (BMSCs) [6]. Stem cells that possess strong self-renewal and multilineage differentiation potential [7], in cases better than non-stem cells. A cell-seeded scaffold is implanted in the patient, and then the cell will produce the new tissue, is the pillars of tissue engineering [8, 9]. Providing seed cell for damaged or lost organs and tissues are the core of tissue engineering [10]. Rapid and complete regeneration of tissue or organs is a very challenging problem because transplanted cells are easily lost in host tissues and have low survival rates [11, 12]. Furthermore, if defective cells migrating to the wound site will lead to a more severe condition [13]. The loss of cellular function at the donor site and uncontrollable differentiation are limitations for the use of stem cell transplantation in regenerative medicine [13–15]. Thus they require the manipulation of cell behavior in vitro and in vivo, including cell proliferation, migration, differentiation, and other cellular processes [16, 17]. The choice of scaffold material, the surface topography of the scaffold, and the additional stimulating factors can manipulate cell behavior [18–21].

To date, several studies have demonstrated that both biochemical and biophysical cues could influence cell behaviors [17, 22, 23]. Different forms of stimulating factors can induce cell proliferation, differentiation, to complete tissue repair, inappropriate ES could cause cell death or no effect [24]. As a result select suitable stimulate factor could maximize the repair effect [25]. Biochemical cues include supplying chemical reagents [26] and performing chemical surface modification on scaffolds [27]. On the one side, adding growth factors, surface-immobilised biosignals, cytokines, and small molecule drugs would be immediately diluted by blood or metabolized by organisms. On the other side, the surface fixation methods of chemical reagents is also not perfect, methods such as silanization or co-precipitation are complexity and low efficiency, requiring more surface treatment to improve connection efficiency [28] and increasing the deposition rate [29]. Biophysical cues involve surface topography, substrate stiffness, compression and stretching, electric or magnetic fields, ultrasound stimulation, and photostimulation [30–34]. Biophysical cues have the advantages of cost-effectiveness, long life, easy to characterize, and high reproducibility, which is facilitated for a large-scale operation. As a biophysical cue, electrical stimulation (ES) has been shown to effectively relieve pain, promote blood circulation, reduce vascular and skeletal muscle tension, and promote reabsorption of edema and joint fluid in the clinic [35]. Also, many studies show that ES could effectively manipulate cell behaviors in vitro and in vivo. For example, Jaatinen et al. [36] demonstrated that the mouse myoblast cell line undergoes dramatic changes in cell morphology, viability, cell structure, and cell adhesion under pulsed monophasic currents. Kumar et al. [37] showed that the cell proliferation and osteogenic differentiation of preosteoblast could favourably be regulated under dynamic electric field conditions. Besides, with the specific parameter of ES, the neurite outgrowth of NSCs could be improved [19], and the differentiation of NSCs could also be matipulated [38]. In the human body, every cell subjected to some form of stimulation, local bioelectrical signals affect cells in a variety of tissues [39]. Indeed, providing ES is a considerable method based on recent research. ES triggers the cells themselves to deliver signals through intrinsic pathways, consequently leads to direct cell activities, including migration, differentiation, and proliferation, etc. Meanwhile, ES can be used synergistically with other techniques, reducing the cost of the whole process, has the potential of alleviating some of the problems that currently prevail in tissue engineering and regenerative medicine [17, 40–42].

Here, we provide an overview of the recent developments of ES in tissue engineering, for better application in regenerative medicine and tissue engineering. The main part of the review is organized into three parts: (1) summarized the conductive materials that can be applied to tissue engineering scaffolds, including types and their advantages and disadvantages. (2) Three methods of providing ES include direct coupling, capacitive coupling, and using an electromagnetic field, and their advantages and disadvantages. (3) The specific regulation of ES on cell behaviors include cell alignment, migration and its underlying mechanisms, proliferation and differentiation. Comprehensively demonstrate the potential that ES has for tissue engineering.

### Different materials and methods to deliver ES

Tissue engineering attempts to imitate the structure and function of the tissues or organs through the use of engineered scaffolds, which optimize the response of cell-biomaterials and mimic the native environment [43]. ES has received considerable attention to influence cellular or tissue behaviors as stimulation factor of tissue engineering [44]. As a bridge to deliver ES, scaffolds need to meet requirements: excellent biocompatibility, prominent electrochemical performance, and no byproduct generation [45].

#### Materials to deliver electrical stimulation

To provide ES for tissue regeneration or functional recovery, a number of materials have been developed to make scaffolds. To date, metallic biomaterials include platinum, and gold is increasingly used in medical applications due to their high mechanical strength, long-term stability, good conductivity, and biocompatibility [46]. However, except noble metals, most metallic materials are easily oxidized, and present weak corrosion resistance [47, 48], the release of metal ion may also cause allergies or carcinogenesis. Surface modification of metallic materials is considered to be an effective method to solve the above problems, include preparation of coatings, and covalent chemical conjugation of bioactive molecules [48]. Conducting polymers, such as poly (3,4-ethylene dioxythiophene), polyaniline, polypyrrole (PPy) have been studied as possible candidates [49]. Although the cracks or delamination of conducting polymers under long-term stimulation restrict the electrode performance, cross-link with a specific agent or in-situ polymerization of the conjugated polymer improving the physical stability while allowing the exploration of their superior properties [50, 51]. Carbon materials such as graphene, carbon nanotubes and carbon aerogels have excellent electrical properties and the ability to be easily bio-functionalized, as well as drug loadings [52–54]. However, the biocompatibility of carbon relative materials is still a significant challenge. Scientists have modified carbon relevant materials to eliminate defects. Depositing and fixing graphene can avoid the damage to cells due to direct contact interaction and wrapping mechanisms [55]. The direct exposure of the carbon nanotubes inside the body presents a high probability of detachment. The above issue was overcome by reinforcing the carbon nanotube patterns inside the biocompatible soft polymer matrix [56]. Table 1 lists the forms of different materials, the electrical conductivity and the advantages and disadvantages.

**Table 1:** The forms, electrical conductivity, advantages and disadvantages of different materials

Materials	Forms	Electrical conductivity	Advantages	Disadvantages
Platinum-gold alloy [46]	Discs	Gold 4.52 × 10^7^ S/m Platinum 9.6 × 10^6^ S/m	High mechanical strength, long-term stability, good biocompatibility and good corrosion resistance	Expensive, cell death caused by ion release
Magnesium (Mg) alloys [48]	Discs	≈2 × 10^7^ S/m	High strength, fracture resistance, good electrical conductivity	Poor biocompatibility; cell death caused by high ion release, and change of local pH
Polypyrrole [57]	Coating	10^2^~ 10^3^ S/cm	Good compatibility and support cell adhesion and growth	Rigid, insoluble and poorly processable
Polyaniline [58]	Film	5~ 10 S/cm	Good environmental stability, low cost, good biocompatibility	Poor mechanical properties and complicated manufacturing methods
Graphene [55]	Coating	10^6^ ~ 10^8^ S/m	Good mechanical properties, easy bio-functionalization and drug loading	Moderate toxicity
Carbon nanotubes [52, 53]	Doping with other materials	1.8 × 10^7^ S/m	High mechanical resilience, good support for active materials, high chemical stability, elasticity	Poor biocompatibility, poor dispersion, insoluble and toxic to the cells

#### Methods to deliver electrical stimulation and its parameters

The methods to deliver ES can mainly divide into three types: direct coupling, capacitive coupling, and using an electromagnetic field.

**Fig. 1:**
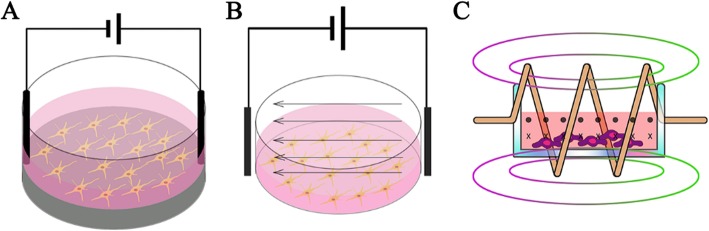
Three ways to deliver electrical stimulation: (**a**) direct coupling, (**b**) capacitive coupling, and (**c**) inductive Reprinted with permission from reference [40]

Direct coupling shows in Fig. 1a. The electrodes are inserted directly into the culture medium and attached to the scaffold to deliver ES. This method is most widely used because of its easy operation. However, drawbacks are apparent such as insufficient biocompatibility of the electrode, contact with the medium lead to temperature rise, pH changes, and the generation of harmful byproducts [59]. Prabhakaran et al. [19] demonstrated that ES applied to the NSCs cultured on the electrospun Poly-L-lactide/polyaniline fibers scaffold exhibited extended neurite outgrowth. Briefly, a silver electrode and a platinum electrode were inserted into the opposite ends of the nanofiber scaffold placed in the medium and connected to constant unipolar trapezoidal pulses. The average length of neurite extended from NSCs with stimulation were nearly 100% higher than the average length of those without ES. In other circumstances, the scaffold is used as substrate-cathode. Stewart et al. [60] induced human NSCs differentiation with a two-electrode device, placing a platinum mesh electrode on top, and using a PPy-coated Au-mylar surface as a working electrode. ES on PPy induced hNSC to differentiate into neurons expressing β-III Tubulin (Tuj1), and a small number of glial cells expressing glial fibrillary acidic proteins, accompanied with longer neurites and significant branches.

Capacitive coupling is more biologically safe compared with direct coupling. As shown in Fig. 1b, two electrodes are placed at opposite ends to provide a uniform electric field to the cells seeded on the scaffold which locates between the electrodes. This system is non-invasive and does not require a conductive scaffold to provide uniform ES. Vaca-Gonzalez et al. [61] used a capacitive coupling system to provide chondrocytes with a 4 mV/cm ES, which significantly increased the cell proliferation. When applied an 8 mV/cm ES, the glycosaminoglycan secreted by chondrocytes remained stable, which is essential to maintain tissue moisture, lubrication, and protection of articular surfaces.

The third type is inductive coupling, as shown in Fig. 1c. Inductive coupling usually uses a controllable electromagnetic field generated by a conductive coil placed around the cell culture system, called pulsed electromagnetic field stimulation (PEMF). The stimulus is transmitted by the pulse to mimic the natural potential transfer in the human body [40]. PEMF provide potential near the target cell, rather than directly apply ES to the cells. The main drawback of PEMF treatment is taking time and resource consumption. For example, some therapies need to last 10 h per day [62] or quite high voltage. Hess et al. [63] created a device using ferromagnetic core covered by the first current-carrying coil, and the culture chamber with connected silicone tube as the second coil. When applied an alternating voltage to the first coil, the second coil only influenced by the electrical potential without any interfering magnetic field or biochemical reactions. The combination of inductive coupling and high sulfated hyaluronan derivative substrate can synergistically stimulate BMSCs, the bone-related proteins (RUNX-2, ALP, OPN) in BMSCs were significantly improved then cells show an osteogenic differentiation. Table 2 lists the advantages and disadvantages of each methods mentioned above.

**Table 2:** The advantages and disadvantages of three methods of providing ES

Methods	Advantages	Disadvantages
Direct coupling [59]	Easy operation	Insufficient biocompatibility of the electrode, contact with the medium lead to temperature rise, pH changes, and the generation of harmful byproducts
Capacitive coupling [64]	More biologically safe	High voltage between the electrodes, longer treatment time
Inductive coupling [62]	Mimic the natural potential transfer in the human body, does not directly touch cells	Tumorigenesis in unexpected area, taking time and resource consumption

The different parameters of ES are quite considerable for regulating cell behavior. ES can provide in the form of monophasic and biphasic, where the waveform has pulse, sinusoidal, square, triangular, and sawtooth pattern. The use of intermittent or continuous stimulation is another parameter. According to the stimulation parameters setup, monophasic stimulation is effective in polarizing the target tissue [65] but may produce reactive oxygen species through the oxidation-reduction process due to the Faradaic reaction at the surface of a metal electrode [66]. Especially in the case of large current pulses delivered at long durations and/or high frequencies, cell damage may result from the Joule heating effect [67]. However, the impact of localized ionic or electrochemical imbalance caused by the small Faradaic impact on the experiment cannot be ignored [66]. On the contrary, biphasic stimulation may be more advantageous since it prevents the charge accumulation, generates lower levels of electrolysis products at the electrodes, outside of the limitations of monophasic stimulation and can be applied for more extended periods and at higher voltages [68–70]. For instance, biphasic stimulation is usually selected in clinical application to stimulate neural tissue, because of the few charge accumulation and toxic by-products and is less likely to cause neuronal loss [69, 71]. Monophasic stimulation can be used for short-term experiments, but long-term applications require biphasic stimulation [72, 73]. Improper stimulation parameters can lead to results that are contrary to experimental expectations. The cell migration rate is positively correlated with the intensity of ES, and is discussed in next section. For stimulus frequency, ES frequency below 1 kHz augment cell proliferation through significantly affected the cell cycle, increasing the proportion of cells and synthesizing DNA [74]. Experiments show that ES frequency above 1 kHz can induce cell differentiation, but must maintain low intensity [75, 76]. High intensity above 100 V/cm can cause cell membrane electroporation, an immediate increase in intracellular Ca^2+^ and reactive oxygen species, then induce cell apoptosis [24, 77]. When high intensity is unavoidable, pulse electrical stimulation should be used to avoid continuous high intensity to reduce damage.

### The effects of electrical stimulation on cell alignment and migration

By participating in tissue formation, tissue regeneration, and wound healing, directed cell migration and alignment can be very beneficial for regenerative medicine [78–81]. The ability to regulate cell migration and alignment would be an invaluable asset for regenerative medicine. The mechanisms underneath the cell alignment and migration are thought to be responsible for these effects, including voltage-gated ion channels, G-protein coupling receptors, integrins, cell polarization, and endogenous electric fields [82]. In literature, ES as the physical methods has attracted much attention because it can activate specific signaling pathways in cells near the cathode or anode and induce cell migration and alignment [83].

#### The effects of electrical stimulation on cell alignment

Numerous methods are capable of inducing cell migration and alignment by external factors, such as the addition of bioactive factors, providing individual surface patterns, and studies have shown that physical methods ES over all other techniques to guide cell migration and alignment [17, 41, 84].

ES has a significant effect on cell alignment and redirect random cells to be aligned, the direction of cell alignment changes gradually as the direction of the ES changes [84–86]. Some types of cell are aligned perpendicular to the direction of the electric field vectors to minimize the field gradient across the cell, such as cardiac adipose tissue-derived progenitor cells [18], endothelial progenitor cells [87], vascular ECs [77], BMSCs [88], adipose-derived stromal cells [89], etc. At the same time, some cells are aligned parallel to the field vectors due to the ES causes rearrangement of the cell cytoskeleton, such as ventricular myocytes [20], cardiomyocytes [86], myoblasts [85], PC-12 cells [90], obsteoblasts [84], etc. The ES intensity usually < 10 V/cm, and the cells aligned better with the ES intensity increased, but the cell activity is relatively decreased [77, 87, 91].

In the presence of 100 mA monophasic ES, the osteoblasts elongation and proliferation were mainly reliant on the ES, whereas the topographical features played a minor role [84]. In our present study [90], perpendicular electrical field vectors from the pattern may reorientate the PC-12 cell alignment, while parallel vectors could further increase neuritis extension compared with perpendicular vectors. On the contrary, some types of cell are aligned perpendicular to the vectors under ES. The murine adipose-derived stromal cells exhibited a constant perpendicular alignment with the field vectors [89]. BMSCs consistent perpendicular alignment to the field vectors [88]. The long axis of cobblestone-like ECs transformed into highly ordered, perpendicular to the field vectors with applied ES, then the cell morphology presents similarly to the inner surface topography of the blood vessel, which shows excellent potential in angiogenesis [77].

#### The effects of electrical stimulation on cell migration

As well as cell alignment, ES plays a significant role in cell migration. The directional migration of cells in response to ES is called electrotaxis [77]. The guiding effect of ES on cell migration also differs depending on the cell type. Among different types of cell, NSCs [92], macrophages [93], mouse neural precursor cells (NPCs) [94], osteoblasts [95] and endothelial progenitor cells [87] toward the cathode [20, 89, 92, 96], BMSCs [97], human dermal fibroblasts [98] and SCs [99, 100] toward the anode. Reversal of the ES polarity reversed the migration direction of cells [87, 92]. The ES intensity can stimulate cell migration from a minimum of 0.1 V/cm to a maximum of 12 V/cm and did not cause any significant damage to cells, did not affect cell phenotype or the differentiation potential [101–103]. At the same time, cells showed gradually increased migration rate and distance with higher ES strength [101, 104, 105].

As NPCs shows a cathodal migration, Liu et al. [103] demonstrated that reversing the ES direction resulted in a reversed direction of NPCs migration. This behavior is beneficial to long-distance migration of neural cells for nerve repair. ES also directs some types of cell migrate to the anode, such as BMSCs [97, 106], human dermal fibroblasts [37] and SCs [99, 100, 107]. Forciniti et al. [100] indicated an increase in average displacement of the SCs to anodal with 0.5 V to 1 V ES, which is of great value in optimizing conductive polymers for different biomedical applications such as nerve repair. Moreover, even different cell lines of the same specie have different responses to ES in the migration. Li et al. [108] demonstrated different cell lines of non-small cell lung cancer cells (H460, and H1299) showed great different in migration direction, while H460 migrated toward cathode, H1299 exhibited anodal migration. This discovery has led to a better understanding of the mechanism of cancer, which is beneficial for cancer therapy.

mASCs exhibited a positive correlation between cell migration rate and ES intensity (from 1 V/cm to 10 V/cm) [89]. The same performance showed in human BMSCs, as the ES intensity is above the physiological level, the migration rate increases while physiological levels of ES do not affect cell motility, this behavior may be essential for maintaining transplanted cells at the lesion site [88, 105]. Different research groups have conflict results in the same cell type. Forciniti et al. [100] showed ES conditions (from 0 to 0.5 V) produced a significant increase in SCs migration rate but did not affect migration direction. Different from others, Li et al. [99] demonstrated the ES did not significantly affect the SCs migration speed, but as the ES intensity increases from 50 mV/mm to 200 mV/mm, the orientation and displacement of the anode migration increase significantly.

#### Mechanism of ES on cell migration

**Fig. 2:**
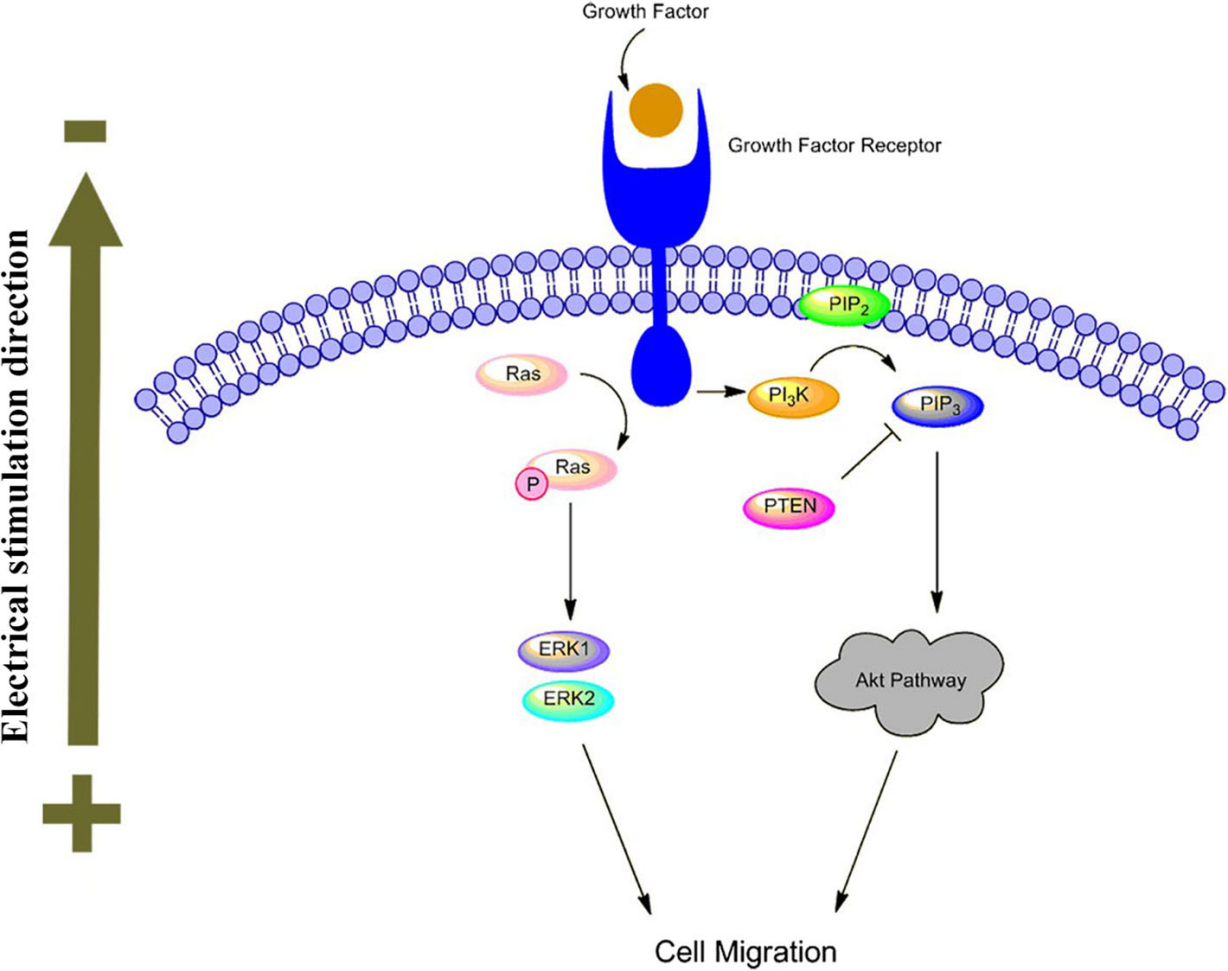
Mechanism of ES-induced cell migration

The specific mechanism leading to electrotaxis is still unclear. Many factors may be included, such as endogenous microenvironment, ion channels, membrane receptors, transport proteins, and competing signal pathways such as Wnt/GSK3β and TGFβ1/ERK/NF-κB [109–111]. According to the literature, the main mechanism involved in ES induced cell migration is shown in Fig. 2.

The importance of epidermal growth factor receptors (EGFR) in ES induced cell migration has attracted the attention of numerous scientists [77, 87, 112]. ES regulates downstream growth factors and cytokine production through a feedback mechanism by EGFR. Asymmetric expression of EGFR in front of and behind cells activates signaling pathways and leads to cell migration [112, 113]. Pilipos et al. [114] demonstrated that blockade of EGFR via erlotinib significantly attenuate NPCs electrotaxis. EGFR activation stimulates several signaling pathways, including MAPK-ERK1/2 and PI3K/Akt pathway. MAPK-ERK1/2 are generally involved in signal transduction from extracellular sources in different signaling pathways. Downstream protein ERK1 and ERK2 are fully activated by phosphorylation of MEK, which is involved in cell migration. Hammerick et al. [89] proved that inhibiting the MAPK pathway leading to migration inhibited suggests the participation of this pathway in mASCs electrotaxis. It has been reported that static monophasic ES can regulate epithelial cell proliferation and migration by activating the MAPK signaling cascade ERK1/2 [115].

The other and the most critical pathway is the PI3K/Akt signaling pathway, which has been mostly explored in the cellular response to ES. ES significantly increase the expression of downstream protein PIP3 and phosphorylation of Akt, at the same time, induce the asymmetric distribution of PIP3 and cytoskeletal proteins migrate toward cathode [113, 116]. Meng et al. [116] exhibited the cathode migration of NPCs under ES needed the activation of the PI3K signal pathway. Pharmacological inhibition or genetic disruption of PI3K/Akt pathway will inhibit the electrotaxis, then applied ES increases Akt phosphorylation and PIP3 fluorescence, demonstrate the importance of the PI3K/Akt pathway in ES driven directed migration of NPCs. PTEN is a phosphatase that inhibits PI3K signaling transduction including Akt. Zhao et al. [117] demonstrated genetic disruption of PTEN within keratinocyte enhanced ES induced ERK and Akt phosphorylation, cell performed a significantly higher directionality compared with controls. This result supports the antagonistic relationship between PI3K and PTEN.

In addition to signal pathways, ion channels such as voltage-gated Ca^2+^ channels is a vital part of membrane polarization and cell response during ES [118]. All living cells have a transmembrane potential. The current induces a flow of ions (Na^+^, Cl^−^, K^+^, Ca^2+^, etc.) by ion channels and transporters [16]. In response to ES, intracellular molecular and transport channel polarization, then ion flow takes place and trigger cytoskeleton changes to direct cell migration, in consequence, the calcium influx contribute to persistent cell migration towards cathode [101]. In the review of Balint et al. [40], the ES effect will be impaired or completely blocked if block the calcium channels, intracellular store and the calmodulin by verapamil and nifedipine, TMB-8, W-7, respectively. Zhao et al. [102] reported that a 115 V/m monophasic ES could cause a calcium influx mediated NPCs mobility improved and cathodal migration.

### The effects of electrical stimulation on cell proliferation and differentiation

In addition to the impact on the migration, ES also plays a significant role in influencing proliferation and guiding differentiation [50, 119, 120]. A major challenge in regenerative medicine is to compensate cells that lost as a result of injury or disease [15]. For instance, after excessive cell loss, most tissues produce scars, such as fibrous collagen scars in the heart, causing an ischemic environment and limiting oxygen delivery [121]. However, the inevitable problems are the difficulty in harvesting sufficient cells for implantation. In these circumstances, the capabilities of proliferation and multilineage differentiation of seed cells have attracted much attention [122]. Table 1 summarizes the specific conditions of ES to induce cell proliferation and differentiation.

#### The effects of electrical stimulation on cell proliferation

Proper ES can promote cell proliferation, usually under continuous stimulation of < 1 V/cm [37, 95, 120]. Within the ES intensity range the cell proliferation rate increases with increasing intensity [95]. Preosteoblasts [123], obsteoblasts [37], unrestricted somatic human stem cells [124], human umbilical vein ECs [125], NSCs [126], human dermal fibroblasts [127] exhibited 0.2 to 1.5 times proliferation, with increasing cellular metabolic activity, and do not affect cell phenotype [123]. High-intensity ES of > 100 V/cm is also favorable for cell proliferation in a short period (< 1 ms) single stimulation, but excessively high intensity leads to cell death [128].

Shao et al. [17] demonstrated that in the presence of 100 mA direct current (DC), the viability of osteoblasts did not affect, and the degree of proliferation increased significantly compared with no stimuli group and other current stimuli groups. This finding indicates that proper ES can be used for bone regeneration and fracture healing. Zhu et al. [126] revealed that compared with the control group, NSCs proliferation was increased 35% with ES, which condition is 100 μA and pulse rates of 100 Hz with 100 μsec duration for 24 h. A possible cause is that ES upregulates the proliferation of cell nuclear antigen, which is a DNA polymerase-associated protein, and activate extracellular signal-regulated kinases 1 and 2 pathways involved in transduction of proliferative signals. Meanwhile, the differentiation potential of NSCs has not been affected by the promotion of proliferation. In the study of Sun et al. [129] SCs underwent a remarkable degree of proliferation under ES (100 mV/cm, 1 h per day) than without ES, especially in day 5. It might because ES promoted SC myelin gene expression and neurotrophin secretion, then led to SCs proliferation. This result indicated the promising potential of ES on peripheral nerve repair and regeneration. Kapeller et al. [119] pointed out that with 1 μA DC stimuli, cardiomyocytes showed better proliferation behavior with no morphological changes in vitro. At the same time, ES also affects matrix metalloproteinases (MMP) in cardiomyocytes, which involved to keep the balance between matrix synthesis and degradation. It shows that ES has a certain positive effect on heart repair.

#### The effects of electrical stimulation on cell differentiation

Cell differentiation based therapy provides a promising approach for regeneration medicine. Short-term (several minutes), low-intensity (0.06~6 V/cm) ES can promote cardiac differentiation of human induced pluripotent stem cells, muscle precursor cells, with the formation of embryoid bodies [130, 131]. Neural progenitor cells, neural precursor cells, and NSCs can differentiate into neurons instead of glial cells under ES [102, 106, 132]. The stimulation intensity is basically < 2 V/cm and sustain more than 7 days. The differential medium can appropriately added with FBS, retinoic acid, and nerve growth factor. ES can induce bone marrow stromal cells, BMSCs, MC3T3-E1 cells osteogenic differentiation instead of cartilage, stimulation intensity should be < 2 V/cm and sustain 14~28 days, the medium need to add dexamethasone in most cases [6, 63, 120, 133–135]. Therefore, the application of ES could provide a valid approach to induce cell differentiation in tissue engineering.

Hernández et al. [131] demonstrated human induced pluripotent stem cells (Foreskin)-2 cell line exhibit cardiac differentiation under ES (65 mV/mm or 200 mV/mm, biphasic current pulses), by significantly increased the expression of cardiac transcription factors *nkx2.5* and *tbx5*, as well as the cardiac contractile muscle proteins ACTC1, TNNT2, MYH7, and MYL7, compared with the unstimulated control group. In the study of Chan et al. [130], human ESCs cultured in the presence of ES (6.6 V/cm, 1 Hz, and 2 ms pulses) showed an increase in the proportion of ventricular-like cardiomyocytes, by significant upregulation of cardiac-specific gene expression including *hcn1, mlc2v, scn5a, serca, kv4.3,* and *gata4*. Matsumoto et al. [135] induced the differentiation of mouse bone marrow stromal cells into neural cells using ES (10 Hz, 100 mV, 2.0 ms, 30 min, rectangular pulse). Neurogenin2 was detected as increased expression through the β-catenin signaling pathway after ES, which is involved in neural differentiation and inhibits astrocytic differentiation. Sun et al. [129] demonstrated ES (100 mV/cm, 1 h) could induce PC-12 cell differentiation into SCs and synaptic elongation even without nerve growth factor treatment, which is a kind of nerve growth regulator that promotes both neuron nutrition and neurite outgrowth.

In the study of Wang et al. [120] ES (200 mV/cm, 1 Hz to 100 kHz, 30 min) promote MC3T3-E1 cells osteogenic differentiation via examined alkaline phosphatase activity. During stimulation, 100 Hz could up-regulate the mRNA levels of collagen I, collagen II, and RUNX2, which are osteosis-related genes. On the other hand, 1 Hz to 10 Hz could improve calcium deposition in the intracellular matrix, which contribute to treat the bone fracture and bone nonunion. Hronik-Tupaj et al. [6] demonstrated ES (20 mV/cm, 60 kHz) improved hMSC osteogenic differentiation potential based on calcium deposition, because of the two-fold increase of alkaline phosphatase and collagen type 1.

Table 3 The specific conditions of ES to induce cell proliferation and differentiation.

**Table 3:** The specific conditions of ES to induce cell proliferation and differentiation

Cell type	Type of ES	Electrical parameters	Major findings
Osteoblast	DC	100 mA	Celluar proliferation, elongation were improved
NSCs	Biphasic current pulses	100 μA, 100 Hz with 100 μsec duration	NSCs proliferation was promoted associating with upregulated neuronal gene expression level and increased microtubule-associated protein 2
Cardiomyocytes	DC	~1 μA	Enhances proliferation with no morphological changes in vitro, modulate the expression of MMPs and TIMPs in vitro and in vivo
Human induced pluripotent stem cells (Foreskin)-2 cell line	Biphasic current pulses	65 mV/mm or 200 mV/mm for 5 min, 1 Hz, and 1 ms pulse width	The cell showed cardiac differentiation with increased the expression of NKX2.5 and TBX5, as well as the proteins ACTC1, TNNT2, MYH7, and MYL7
Human ESCs	N/A	6.6 V/cm, 1 Hz, and 2 ms pulses	Upregulation of gene expression including HCN1, MLC2V, SCN5A, SERCA, Kv4.3, and GATA4; cellular elongation, and an increase in the proportion of ventricular-like hESC-derived cardiomyocytes
Mouse bone marrow stromal cells	Rectangular pulse	100 mV, 10 Hz, 2.0 ms, 30 min	Induced the differentiation of mouse BMSCs into neural cells with enhanced neurogenin2 (Ngn2) expression
SCs & PC12 cell	N/A	100 mV/cm, 1 h	Promote SCs proliferation, and promoted PC12 cell differentiation into SCs and axonal extension
Mouse embryonic osteoblast precursors Mc-3 T3-E1	Rectangular pulses	200 mV/cm, 1 Hz to 100 kHz, 30 min	100 Hz could up-regulate the mRNA levels of collagen I, collagen II and Runx2, accelerate cells differentiation and proliferation, down-regulate the mRNA levels of osteopontin (OPN). 1 Hz to 10 Hz could improve calcium deposition in the intracellular matrix.
BMSCs	N/A	20 mV/cm, 60 kHz	An increase in ALP and col1 transcript, and NADH, FAD, lipofuscin was detected, improved hMSC differentiation potential to bone

## Summary

In this review, we have highlighted the effects of ES as a physical stimulator on cellular behavior for the purposes of applying to regenerative medicine and tissue engineering. In most cases, ES facilitates cell proliferation and differentiation, enhance cell cathode migration and alignment to field vectors, and mainly through EGFR, PI3K and Ca^2+^ related mechanism. ES can be delivered through tissue engineering scaffolds made of metallic biomaterials, conducting polymers, or carbon materials, the main methods are direct coupling, capacitive coupling, and using an electromagnetic field.

In the future, the combination of specific material/structure and ES will offer many advantages over other types of stimulations and allow for precise cellular regulation. Developing safe and effective partition-type scaffold combine with ES that can distinguish different areas to perform different stimulation still requires some challenges to be overcome. Although still in its early stages, the field of ES is rapidly evolving, and new next-generation regenerative medicine and tissue engineering will make it possible to take advantage of ES. Studying the low risk ES method for wearable application is also a future direction. With increasing research, electrical stimuli have the potential to play a significant role in tissue engineering and regenerative medicine.

## Data Availability

Not applicable.
